# Temporal Changes in Rat Liver Gene Expression after Acute Cadmium and Chromium Exposure

**DOI:** 10.1371/journal.pone.0127327

**Published:** 2015-05-19

**Authors:** Michael S. Madejczyk, Christine E. Baer, William E. Dennis, Valerie C. Minarchick, Stephen S. Leonard, David A. Jackson, Jonathan D. Stallings, John A. Lewis

**Affiliations:** 1 ORISE Postdoctoral Fellow at the US Army Center for Environmental Health Research, Fort Detrick, MD, United States of America; 2 Excet, Inc., Fort Detrick, MD, United States of America; 3 US Army Center for Environmental Health Research, Fort Detrick, MD, United States of America; 4 National Institute for Occupational Safety and Health, Morgantown, WV, United States of America; North Carolina A&T State University, UNITED STATES

## Abstract

U.S. Service Members and civilians are at risk of exposure to a variety of environmental health hazards throughout their normal duty activities and in industrial occupations. Metals are widely used in large quantities in a number of industrial processes and are a common environmental toxicant, which increases the possibility of being exposed at toxic levels. While metal toxicity has been widely studied, the exact mechanisms of toxicity remain unclear. In order to further elucidate these mechanisms and identify candidate biomarkers, rats were exposed via a single intraperitoneal injection to three concentrations of CdCl_2_ and Na_2_Cr_2_O_7_, with livers harvested at 1, 3, or 7 days after exposure. Cd and Cr accumulated in the liver at 1 day post exposure. Cd levels remained elevated over the length of the experiment, while Cr levels declined. Metal exposures induced ROS, including hydroxyl radical (•OH), resulting in DNA strand breaks and lipid peroxidation. Interestingly, ROS and cellular damage appeared to increase with time post-exposure in both metals, despite declines in Cr levels. Differentially expressed genes were identified via microarray analysis. Both metals perturbed gene expression in pathways related to oxidative stress, metabolism, DNA damage, cell cycle, and inflammatory response. This work provides insight into the temporal effects and mechanistic pathways involved in acute metal intoxication, leading to the identification of candidate biomarkers.

## Introduction

Chromium (Cr) and cadmium (Cd) are widely distributed and some of the most utilized metals in industry, thus posing occupational and environmental exposure risks to both the general population and military personnel. Exposure to these metals can occur through contact with contaminated soil, air, water, and food as a result of manufacturing, pharmaceutical, industrial processes or environmental contamination. Cr is extensively used for stainless steel production, chrome plating, and as an anti-corrosive, which can lead to increased occupational exposures. Toxic Cr exposures may result from the ingestion or inhalation of dusts generated while refurbishing metal parts (e.g., Cr coated steel from aircraft) or from bulk materials present at industrial sites, such as what occurred at the Qarmat Ali water treatment facility in Iraq [1]. Cd exposure can occur as a result of mining, metal processing, welding, burning fuels, the production and use of phosphate fertilizers, leaching of metal waste, and smoking [2]. Due in part to their abundance and wide-spread use, they were also highly ranked in an industrial chemical prioritization and hazard analysis conducted by the Naval Research Laboratory [3].

The liver plays important roles in metal homeostasis and detoxification. A major hepatic function involves the uptake of ingested metals from portal blood before they are able to distribute to other organs (i.e., first pass clearance). Once absorbed, the metal ions are quickly bound to intracellular ligands. Some are specific metal-binding ligands, which act as metal chaperones to guide metals to their appropriate destination within the cell, a few of which have been characterized at the molecular level [4–7]. Other less specific ligands also play a more general role in metal sequestration and disposition, including proteins such as metallothionein (MT), ferritin, glutathione (GSH), and small molecules such as citrate and ascorbate, and amino acids. Bound metals may be shuttled to organelles for storage, incorporated into metalloproteins (e.g., manganese into superoxide dismutase [SOD] or zinc [Zn] into MT), and distributed into the bloodstream for delivery to other tissues, or excretion into bile [8]. Cd and Cr can also utilize these same pathways. For example, Cd is a substrate for the divalent cation uptake transporter DMT1, and once inside the cell it can substitute for Zn on MT or iron on ferritin [9,10]. Substitution of the wrong metal cofactor into metalloproteins can lead to a disruption of normal function, such as is seen when Cd replaces Zn in the DNA repair protein XPA [11]. Another important role of the liver is the excretion of metals and metal complexes into bile. Biliary excretion acts as a primary or secondary pathway for the elimination of a number of essential and toxic metals, including copper, manganese, mercury, lead, Cd and Cr. Due to its important roles in metal metabolism, distribution and elimination, the liver is also susceptible to damage if it’s homeostatic and detoxification mechanisms are impaired or overwhelmed.

Exposure to Cd and Cr can lead to similar adverse health effects in target organs such as the liver, kidney, and lungs, although they are thought to act via different mechanisms and biochemical pathways. Numerous animal studies have also reported Cd and Cr-induced liver damage [12–17]. Cd is known to accumulate in the liver and its half-life for excretion has been reported to range from 4–19 years [18], while the urinary excretion half-life for Cr in humans is approximately 39 h [19]. A major contributor to tissue damage is oxidative stress, which is induced after exposure to both metals through the production of reactive oxygen species (ROS); however the mechanisms by which they are produced differ. Studies suggest that hexavalent chromium [Cr(VI)] is reduced to its lower oxidation states, which result in free radical generation, through Fenton-like reactions [20,21]. Cadmium is thought to act via the inhibition of antioxidant enzymes and the depletion of glutathione (GSH) leading to ROS production [22]. The International Agency for Research on Cancer (IARC) has classified both Cd and Cr as known human carcinogens [23]. Although Cr can interact directly with DNA forming Cr-DNA adducts, Cd is thought to induce DNA damage by inhibiting repair enzymes [24].

The use of toxicogenomics technologies to study the transcriptome, proteome and metabolome is beginning to provide insight into the mechanisms and molecular pathways that are involved in metal intoxication, the tissues’ adaptive response, and the development of adverse health effects. The use of these technologies allows for a global approach to better understand the cellular and molecular pathways involved in metal induced cellular damage, to visualize potential cross-talk between pathways, and to compare cellular responses to different stressors. For example, previous work in our laboratory compared the gene response of a rat-liver derived cell line to Cd, Cr, and Ni [25]. We were able to identify global responses that were common to all three metals, such as the induction of oxidative stress, as well as to identify the individual gene responses unique to each metal. A greater understanding of these effects coupled with linking them to known markers of health effects will provide a useful tool for biomarker discovery, screening for exposure to environmental pollutants and/or predicting the risk of disease.

To gain a better understanding of the response in the liver after metal exposure, we utilized traditional toxicity assays as well as microarray analysis of the liver at 1, 3, or 7 days after a single exposure to CdCl_2_ or Na_2_Cr_2_O_7_. Both metals accumulated dose-dependently in the liver and generated ROS, including hydroxyl radical (•OH). Interestingly, the greatest amount of ROS was induced well after the metal exposure. This would be expected as Cd accumulated in the liver, however, ROS continued to increase as the amount of Cr and differences in gene expression in the liver decreased. In addition, we identified 667 and 879 probe sets, for Cd and Cr respectively, which were differentially expressed over the 7 day period. The differentially expressed gene lists for the Cd and Cr exposures were enriched for oxidative stress, metabolism, DNA damage, cell cycle, and inflammatory response pathways.

## Materials and Methods

### Animals

Research was conducted in compliance with the Animal Welfare Act, and other Federal statutes and regulations relating to animals and experiments involving animals and adheres to principles stated in the Guide for the Care and Use of Laboratory Animals (NRC 2011) in facilities that are fully accredited by the Association for Assessment and Accreditation of Laboratory Animal Care, International. The animal facilities are specific pathogen-free and environmentally controlled. All animal procedures used during the study were reviewed and approved by the National Institute for Occupational Safety and Health (NIOSH) animal care and use committee.

Male Sprague-Dawley rats [Hla:(SD) CVF] were purchased from Hilltop Lab Animals (Scottdale, PA), weighing 250–300 g and free of viral pathogens, parasites, mycoplasmas, *Helicobacter*, and CAR *Bacillus*, and were used for all exposures. The rats were acclimated for at least 6 days after arrival, were housed in ventilated polycarbonate cages on a mixture of Alpha-Dri cellulose chips and hardwood Beta-chips bedding, and were provided HEPA-filtered air, irradiated Telad 2918 diet, and tap water *ad libitum*. Chemicals and reagents were obtained from Sigma-Aldrich Chemical Co. (St. Louis, MO), or J.T. Baker (Philipsburg, NJ).

### Animal exposures

Rats received either vehicle (sterile saline) or metals in vehicle by intraperitoneal injection, and organs, blood and tissues were harvested at 1, 3 and 7 days post injection. Intraperitoneal injection was chosen as the most direct method of modeling metal exposure as it will tend to maximize toxicant exposure to target organs, including the liver. Rats were exposed via a single injection to 0.5, 1.25 and 2.5 mg/kg body weight (BW) CdCl_2_ or 5, 10 and 20 mg/kg BW Na_2_Cr_2_O_7_. Previous studies have demonstrated the use of intraperitoneal injections of CdCl_2_ [26–34] and Na_2_CrO_4_ [35–38] in these dose ranges to induce tissue injury. Exposure times of 1, 3 and 7 days were chosen to investigate the acute effects of metal exposures and were based on previous investigations for changes in oxidative stress and recovery time [39,40]. Rats were deeply anesthetized at 1, 3 and 7 days post-exposure with an intraperitoneal injection of sodium pentobarbital (100 mg/kg BW; Butler Co., Columbus, OH), exsanguinated by severing the abdominal aorta and tissue collected.

### Determination of liver metal content by ICP-MS

The Cd and Cr content of liver tissue were determined by Inductively Coupled Plasma-Mass Spectrometry (ICP-MS). Liver samples were prepared using a Mars 5 microwave digestion system (CEM, Matthews, NC). Tissue samples (0.1–0.5 g) were placed in 5 mL of nitric acid (Optima grade, Fisher Chemical Co., Pittsburgh, PA) inside a MARSXpress (CEM, Matthews, NC) digestion vessel. Samples were digested using the following program: Step 1) 25% power (400 W), ramp time 20 min, hold time 10 min, max temperature 50°C. Step 2) 50% power, ramp 20 min, hold 10 min, max temperature 120°C. Step 3) 100% power, ramp 20 min, hold 10 min, max temperature 200°C. Following digestion the samples were allowed to cool and then diluted with deionized water to a final acid concentration of 10%. A digestion spike and digestion blank were included in each batch of samples.

Samples were analyzed using an Agilent 7500ce ICP-MS (Santa Clara, CA) using Helium collision mode. Stock solutions containing the elements of interest were purchased from Spex Certiprep (Metuchen, NJ). The instrument was calibrated using standards prepared in 10% nitric acid at concentrations that bracketed the expected concentration range of the samples. Three replicates were analyzed for each metal with varying integration times (1.5 seconds for Cd, 0.5 seconds for Cr). Internal metal standards were added using a mixing tee prior to the introduction of sample to the plasma. The lowest calibration standard analyzed was 1 μg/L, which was used as the limit of detection. Peak intensities were measured against internal standards. The ratios were plotted against concentrations and linear regression analysis performed on the internal standard ratio data. Calculations for the concentration of metals in the tissue were determined using the following equation: concentration in solution (μg/L) * volume of sample (L) / weight of sample (g) = weight of metal / weight of sample (μg/g).

### Hydroxyl radical measurements

Electron spin resonance (ESR) spin trapping was used to detect short-lived free radical intermediates. Flash frozen tissue slices (~100 mg), were homogenized in 1 mL sodium phosphate buffer (50 mM) containing the protease inhibitors: leupeptin (10 μg/mL), phenylmethylsulfonyl fluoride (100 μg/mL), dithiothreitol (1 mM), and trypsin inhibitor (20 μg/mL). The homogenate was immediately reacted with 4-hydroxy-2,2,6,6,- tetramethyl-piperidine-N-oxyl (hydroxy-TEMPO; 0.1 mM) with or without dimethylthiourea (DMTU; 50 mM). All ESR measurements were conducted using a Bruker EMX spectrometer (Bruker Instruments Inc., Billerica, MA) and a quartz flat cell assembly. Hyperfine couplings were measured (to 0.1 G) directly from magnetic field separation using potassium tetraperoxochromate (K_3_CrO_8_) and 1,1-diphenyl-2-picrylhydrazyl (DPPH) as reference standards [41,42]. The Acquisit program was used for data acquisitions and analyses (Bruker Instruments Inc., Billerica, MA). The ESR peak height was used as a relative measure of the amount of free radicals present in the tissue. The contribution of •OH was estimated by taking the difference in peak heights with or without DMTU.

### Lipid peroxidation

Lipid peroxidation of liver tissue was measured using a colorimetric assay for malondialdehyde according to the manufacturer’s instructions (LPO-586, Oxis International Inc. Portland, OR). Briefly, tissue was perfused and washed with ice cold phosphate-buffered saline (PBS; pH 7.4; Ca^2+^ and Mg^2+^ free) to remove blood from sample. Tissue was then homogenized after the addition of 0.5 μL butylated hydroxytoluene to prevent any sample oxidation during processing. A reaction mixture contained 0.1 mg/mL tissue in a total volume of 1.0 mL PBS. A Fenton reaction, composed of FeSO_4_ (1mM), H_2_O_2_ (1 mM) and 0.1 mg/mL tissue, was also carried out as a positive control. The mixtures were exposed for 1 h in a shaking water bath at 37°C. The samples were centrifuged 3000 x *g* at 4°C for 10 min and the supernatant removed for measurement. The absorbance of the supernatant was measured at 586 nm as a measure of lipid peroxidation based on the reaction of a chromogenic reagent with malonaldehyde at 45°C.

### Comet assay

The Comet assay was performed using methods described in the Trevigen CometAssay kit (Trevigen, Inc., Gaithersburg, MD). A typical reaction mixture contained 0.1 mg/mL liver tissue brought to a total volume of 1.0 mL in ice cold PBS containing 20 mM EDTA. All steps were performed in the dark or low light conditions. Briefly, tissue was minced into very small pieces using a cell chopper and let stand for 5 minutes. The resulting cell suspension was then centrifuged and cells placed in ice cold PBS at 1 x 10^5^ cells/mL. The cells were combined with low melting point agarose, and then pipetted onto a Comet slide. The slide was placed in a refrigerator for 30 min, immersed in the supplied lysis solution, chilled for 60 min, and then immersed in alkaline solution (300 mM NaOH) for 55 min. Slides were placed in a horizontal electrophoresis chamber for 40 min at 300 mA. Slides were washed and SYBR Green stain was added. Slides were visualized using fluorescence microscopy, with an image capturing system (Olympus AX70 and sample PCI, Compix, Cranberry Township, PA). A minimum of 50 cells was scored for each sample at 400X magnification. The distance between the edge of the head and the end of the tail was measured using an automated image analysis system (Optimas 6.51, Media Cybernetics Inc., Silver Spring, MD) [43,44]. Only two comet assays from the Cd control and low dose groups were available for analysis, while there were at least three for all other conditions.

### Measurement of H_2_O_2_ in liver tissue

Liver tissue (100 mg) was homogenized in PBS buffer. Homogenization consists of a 20 s burst with a Tissue Tearer (Biospec, Racine, WI) followed by 50 strokes with a tissue grinder. An aliquot of homogenate (100 μl) was taken for protein measurement. The homogenate was centrifuged at 2,500 x *g* for 15 min at 4°C and the supernatant was collected. H_2_O_2_ content was measured by the Oxis Bioxytech H_2_O_2_-560 Quantitative Hydrogen Peroxide Assay Kit (OXIS International, Portland, OR). This assay is based on the oxidation of ferrous ions (Fe^2+^) to ferric ions (Fe^3+^) by H_2_O_2_ under acidic conditions. The ferric ion binds with the indicator dye xylenol orange {3,3-bis[N,N-di(carboxymethyl)-aminomethyl]-o-cresoisulfone-phthalein sodium salt} to form a stable colored complex that was measured at 560 nm using a Spectra Max 250 multi-well plate reader (Molecular Devices, Sunnyvale, CA, USA).

### Statistical analysis

SigmaPlot 11 software (Systat Software, Inc., San Jose, CA) was used for statistical analysis unless otherwise stated. Results are presented as means ± standard error. Statistical comparisons were made using analysis of variance (ANOVA) followed by a Student’s *t*-test. A value of *p* ≤ 0.05 was considered significant.

### Microarray preparation and processing

Total RNA was extracted using Trizol solution (Invitrogen, Grand Island, NY) from samples of liver tissue flash frozen in liquid N_2_, followed by purification with an RNeasy Mini Kit (Qiagen, Germantown, MD) to remove residual salts and organic solvents per the manufacturer’s instructions. The 1.25 and 10 mg/kg body weight doses, for Cd and Cr respectively, were selected for analysis. The doses for microarray analysis were chosen by examining via quantitative PCR the expression of a panel of genes we have observed to respond to metal exposure (*Mt1a*, *Klf5*, *Hmox1*, *Emp1*, *C5*, *Atf3*, and *Fabp1*) [25]. The medium dose for Cd exposure was chosen as there were no significant changes in gene expression between the medium and high doses (Figure A in S1 File). A good correlation was observed between qPCR gene expression values and microarray platform results (r^2^ = 0.87; Table A in S1 File). The largest changes in gene expression after Cr exposure occurred in the high dose group and this dose was therefore selected for this analysis. RNA quality and quantity were determined using the Agilent Bioanalyzer Series II RNA 6000 Nano LabChip Kit on a 2100 Bioanalyzer (Agilent, Palo Alto, CA). Using the Affymetrix 3’IVT kit, cDNA and labeled cRNA were prepared, washed, stained, and hybridized onto the Affymetrix Rat Genome 230 2.0 array and scanned on an Affymetrix GeneChip Scanner 3000 (Santa Clara, CA) per the manufacturer’s instructions.

### Microarray data analysis

Microarray data was processed for background adjustment, normalization, and summarization by the Robust Multi-Array Averaging method (RMA; Irizarry, 2003) using Partek Genomic Suite (GS) 6.6 (Partek Inc., St. Louis, MO). The raw data files were uploaded to the NCBI Gene Expression Omnibus (accession number GSE65198). The microarray data was examined for outliers using a principal component analysis (PCA) in Partek GS. Pairwise correlation analysis between replicates and inter-replicate dot plots of all probe sets were performed to verify reproducibility and identify outliers. Replicates with an R^2^>0.95 and no gross deviations from linearity on the dot plot were accepted. A present, absent, or marginal detection call for each probe set was determined using GeneChip Operating Software (Affymetrix, Santa Clara, CA), and only probe sets with a present detection call for all replicate samples in at least one condition were retained for analysis.

An ANOVA was performed to determine which genes were differentially expressed. The 15,870 probe sets for Cd and 16,110 for Cr that met the present detection criteria were analyzed via ANOVA with contrasts for each exposure versus the pooled controls in Partek GS to determine which genes were differentially expressed due to treatment. One of the day 1 medium dose samples for Cd clustered tightly in the PCA with the unexposed controls. Upon further analysis, this replicate was also found to not have accumulated Cd and was excluded in the analysis. Probe sets were retained for bioinformatic analysis with a Benjamini and Hochberg FDR ≤ 0.05 [45], and a 1.8 or greater fold change from control [46]. On days 3 and 7 there were few or no significant gene changes observed for either metal using this selection cutoff, therefore a less stringent criteria consisting of a ≥1.8-fold change and an unadjusted *p*-value ≤ 0.05 were used to identify differentially expressed genes that may still be changing at these later time points [47].

Ingenuity Pathway Analysis (IPA) software (Ingenuity Systems, www.ingenuity.com) and the Database for Annotation, Visualization and Integrated Discovery (DAVID; version 6.7) were used to explore the biological implications of the data [48,49]. IPA Core analyses were performed using the Rat Genome 230 2.0 array as the reference set for transcriptomics data, with all other default settings selected. We considered IPA canonical pathways, functions, and other enrichments statistically significant with a *p*-value ≤ 0.05 and involving more than two molecules, and transcriptional regulator activation was considered significant with an activation z-score ≤-2 or ≥2 and a *p*-value ≤ 0.05. DAVID Functional Annotation Clustering was also used to identify and bin the probe sets [48,49]. Probe set lists from both exposures were analyzed using the present lists for each exposure as background [49]. Clustering was based on Gene Ontology Biological Process terms and KEGG pathways with default settings, except for the initial and final group settings set to five.

## Results

### Metal accumulation

**Fig 1:**
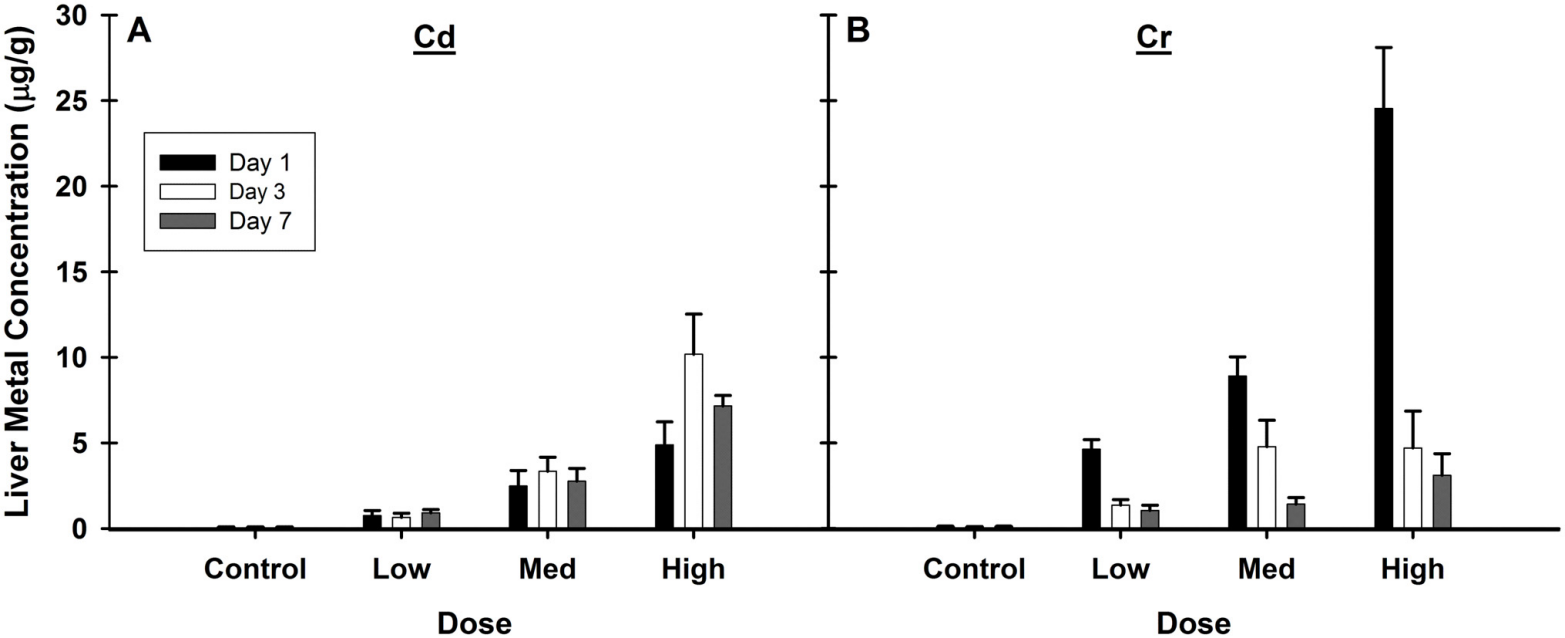
Elevated concentrations of Cd and Cr in the liver 1, 3, and 7 days after a single exposure. Levels of Cd (A) and Cr (B) were determined via ICP-MS. Both Cd and Cr accumulated in a dose-dependent manner at 24 h. Cd levels remained elevated for all doses on days 3 and 7 post-exposure. Cr levels rapidly declined between days 1 and 3, and this trend continued at the low and medium doses, but in the high dose remained at day 3 levels on day 7. Values are mean ±SE, n = 5–7 animals per group.

The consequences of these single exposures to Cd and Cr were long lived and could be observed at the molecular and transcriptional levels. Confirmation of Cd and Cr accumulation in the liver was obtained via ICP-MS (Fig 1). As expected, there was a dose-dependent increase in liver Cd concentration on day 1 (Fig 1A) and the Cd levels remained elevated through day 7. There was no significant difference in Cd levels between days of the same dose. There was also a dose-dependent accumulation of Cr on day 1 (Fig 1B). The high dose Cr accumulation was consistent with a previous experiment that also utilized the same method of exposure [50]. In contrast to the pattern of Cd accumulation, Cr levels rapidly declined from their peak at day 1 and were substantially reduced on days 3 and 7, indicative of its rapid clearance from the body. The hepatic half-life of Cr was determined by fitting the data to a first-order exponential decay model using SigmaPlot 11 (Systat Software, Inc., San Jose, CA), yielding estimated half-lives of 20.1 h (range 13–43 h) at the high dose and 34.3 h at the low dose (range 27–47 h), which are slightly lower but in the same magnitude as the previously published urinary half-life of 39 h for humans [19].

### ROS

**Fig 2:**
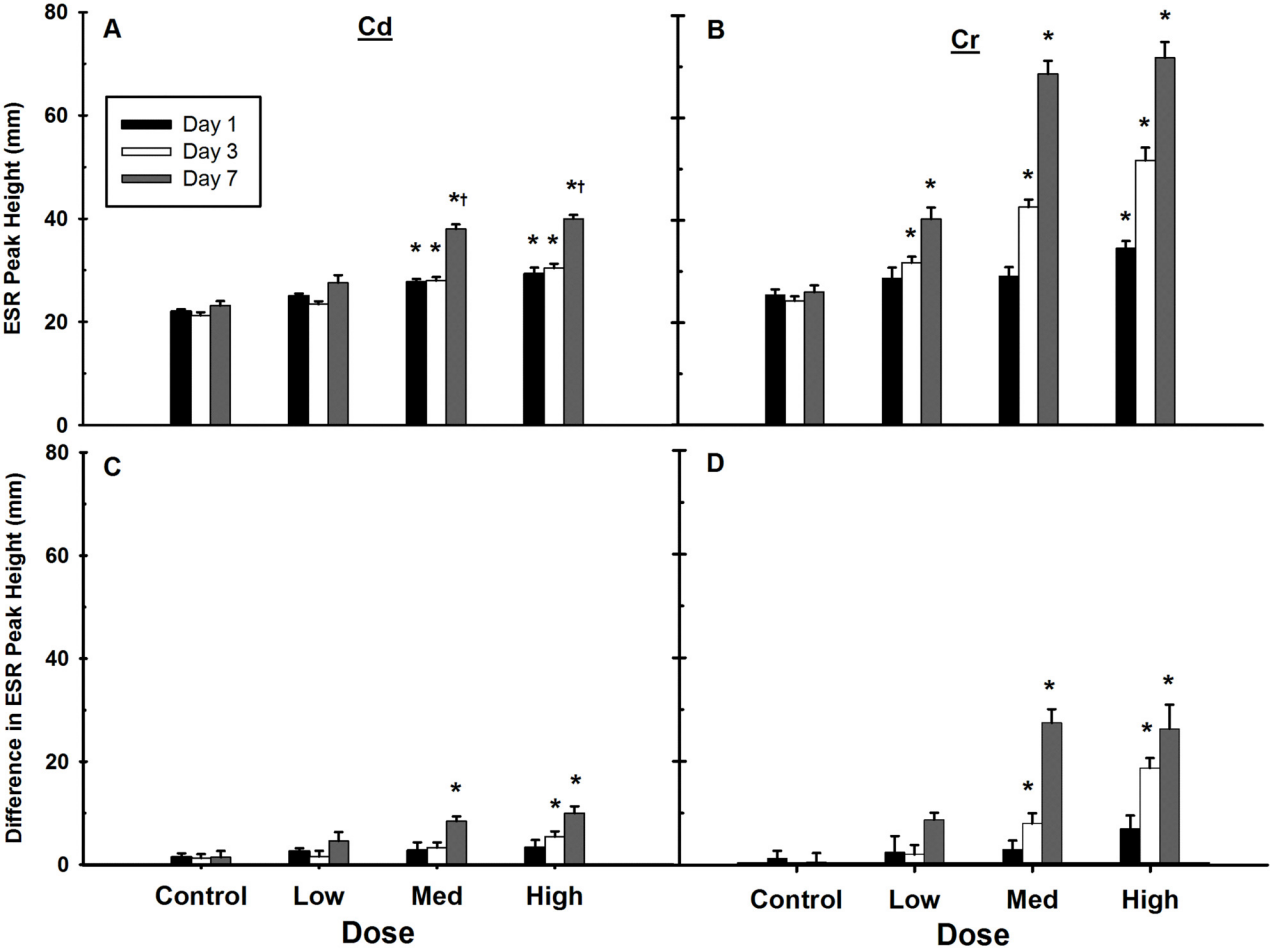
Delayed induction of free radicals in the liver after Cd and Cr exposure. Liver tissue slices from Cd (A and C) and Cr (B and D) exposed animals were incubated with hydroxy-TEMPO with or without the •OH scavenger DMTU, and examined via ESR. Total free radical levels dose-dependently increased for both metal exposures 1, 3 and 7 days post-injection (A and B). An indication of the contribution of •OH to total ROS was determined by examining the differences in ESR peak height after the addition of DTMU (C and D). Cr exposure dose-dependently increased •OH production at the mid and high doses on days 3 and 7. Values are mean ±SE, n = 6–7 animals per group. *Significantly different from control on each day, *p*<0.05. ^†^Significantly different from other days in the same dose.

Metals are well known to induce ROS via multiple mechanisms. Key molecules in the ROS family include the superoxide anion radical, which can be dismutated to form H_2_O_2_, and the highly reactive •OH [51]. Here, the induction of free radicals was monitored using ESR. Liver tissue homogenates were incubated with hydroxy-TEMPO with or without the addition of the •OH scavenger DMTU [52]. The ESR peak height was used as a relative measure of the amount of free radicals present in the tissue [53]. The medium and high doses of Cd induced similar increases in liver ESR peak heights, reflecting an increase in the total amount of free radical oxygen species present (Fig 2A). At these doses, the ROS remained elevated out to at least seven days, similar to the Cd accumulation seen in Fig 2A. ROS were greatest on day 7, and were markedly higher than those observed on days 1 and 3.

Here, we used the difference in ESR peak heights after the addition of DMTU as an indirect measure of the contribution of •OH to the increase in intracellular radicals. The •OH is known to play a role in but is not the sole mediator of Cr(VI)-induced DNA damage when observed in cell culture [54]; however, there is little evidence for its production after Cd or Cr exposures in animal models [55,56]. Analogous to total radicals after Cd exposure, •OH also peaked at seven days post-exposure at the medium and high doses (Fig 2C). In Cr exposed livers ESR peak heights trended upwards in time and dose-dependent manners for both total free radicals (Fig 2B) and •OH (Fig 2D). Within each dose level, the amount of total free radicals increased in a time-dependent manner (Fig 2B). A dose-dependent increase was also observed, with an apparent saturation in free radical formation on day 7. A similar time-dependent effect was also observed when the contribution of •OH was estimated with the addition of DMTU (Fig 2D). The increase in •OH accounts for the majority of the increased radicals observed in Fig 2A and 2B.

**Fig 3:**
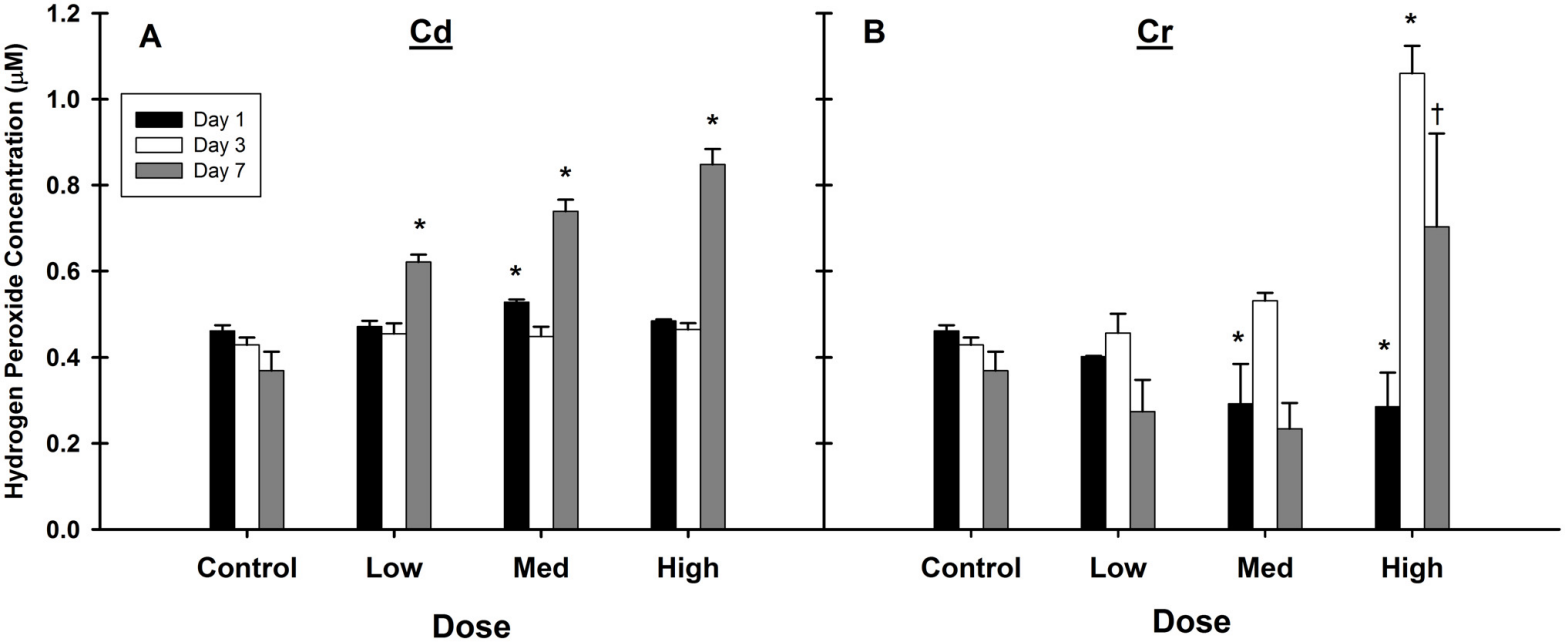
Altered H_2_O_2_ levels in the liver occur well after Cd or Cr exposure. H_**2**_O_**2**_ concentrations were monitored via a colorimetric assay. All three doses of Cd (A) increased H_**2**_O_**2**_ concentrations on day 7 in a dose-dependent manner. Only the high dose of Cr (B) led to increased H_**2**_O_**2**_ concentrations on days 3 and 7. Values are mean ±SE, n = 6–7 animals per group. *Significantly different from control on each day, *p*<0.05. ^†^ *p* = 0.053.

In addition to the ESR free radical measurements, the amount of H_2_O_2_ present in the liver was measured using a colorimetric method. Intracellular H_2_O_2_, which under normal physiological conditions can range from 0.001–0.7 μM, plays important roles in cellular signaling and host defense [57,58]; however, excess H_2_O_2_ can also serve as a source of damaging oxygen radicals in the cell. In livers from Cd treated animals (Fig 3A), H_2_O_2_ levels remained essentially unchanged from controls on days 1 and 3. The single exception was a small, but significant increase at the medium dose on day 1. Although this data point was statistically significant, it is unclear why it was the only dose elevated. However, there was a dose-dependent increase in H_2_O_2_ concentration on day 7 (Fig 3A). H_2_O_2_ levels were significantly lower on day 1 at the medium and high doses after Cr exposure (Fig 3B). At the high dose of Cr, the concentration of H_2_O_2_ spiked on days 3 and 7. The delayed ROS induction may not be a direct effect of metal exposure, as •OH and H_2_O_2_ levels were highest on day 7, while metal levels are highest on days 1–3.

### Lipid and DNA damage

**Fig 4:**
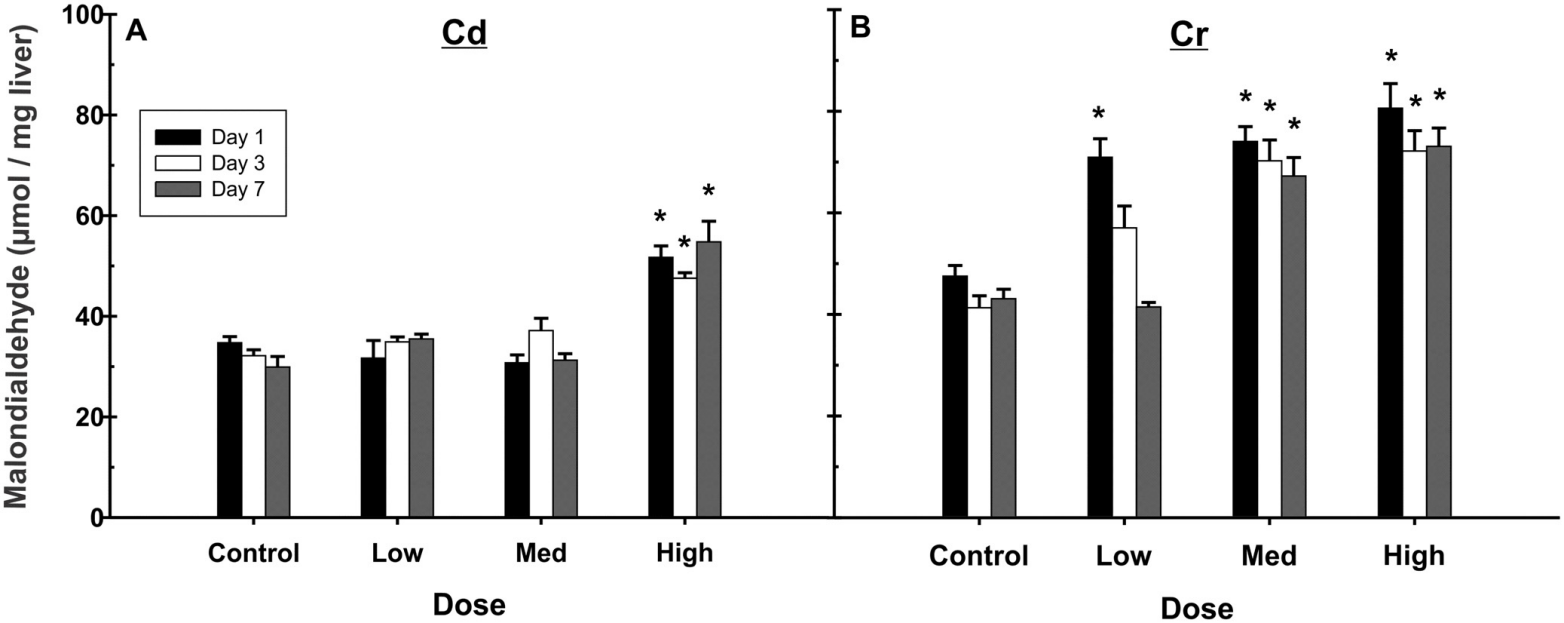
Exposure to Cd and Cr increased lipid peroxidation in the liver. Cd (A) induced a moderate amount of malondialdehyde (MDA) formation at the highest dose, and this amount was sustained through the duration of the study. Cr (B) induced MDA formation at all concentrations tested. At the low dose, MDA returned to control levels over time. However, at the medium and high doses of Cr, MDA levels were elevated for the duration of the experiment. Data are expressed as mean ±SE, n = 6–7 animals per group. *Significantly different from control on each day, *p*<0.05.

Toxicological endpoints of metal exposure and ROS induction were also investigated. Hydroxyl and other radical species can interact with the lipids composing cellular membranes, leading to cellular damage [59], and both Cd and Cr have been reported to induce hepatic lipid peroxidation *in vivo* [10,15,50,60,61]. Radical induced lipid damage was estimated using a colorimetric assay for the lipoxidation end-product malondialdehyde (MDA). Levels of MDA were unchanged in Cd treated animals at the low and medium doses, but were elevated ~1.5–1.8 fold at the high dose (Fig 4A). Cr treatment induced MDA formation at all concentrations on day 1 (Fig 4B), when the metal concentration was highest. At the low dose of Cr, MDA returned to control levels by day 7, while it remained elevated for the duration of the study in the medium and high doses.

**Fig 5:**
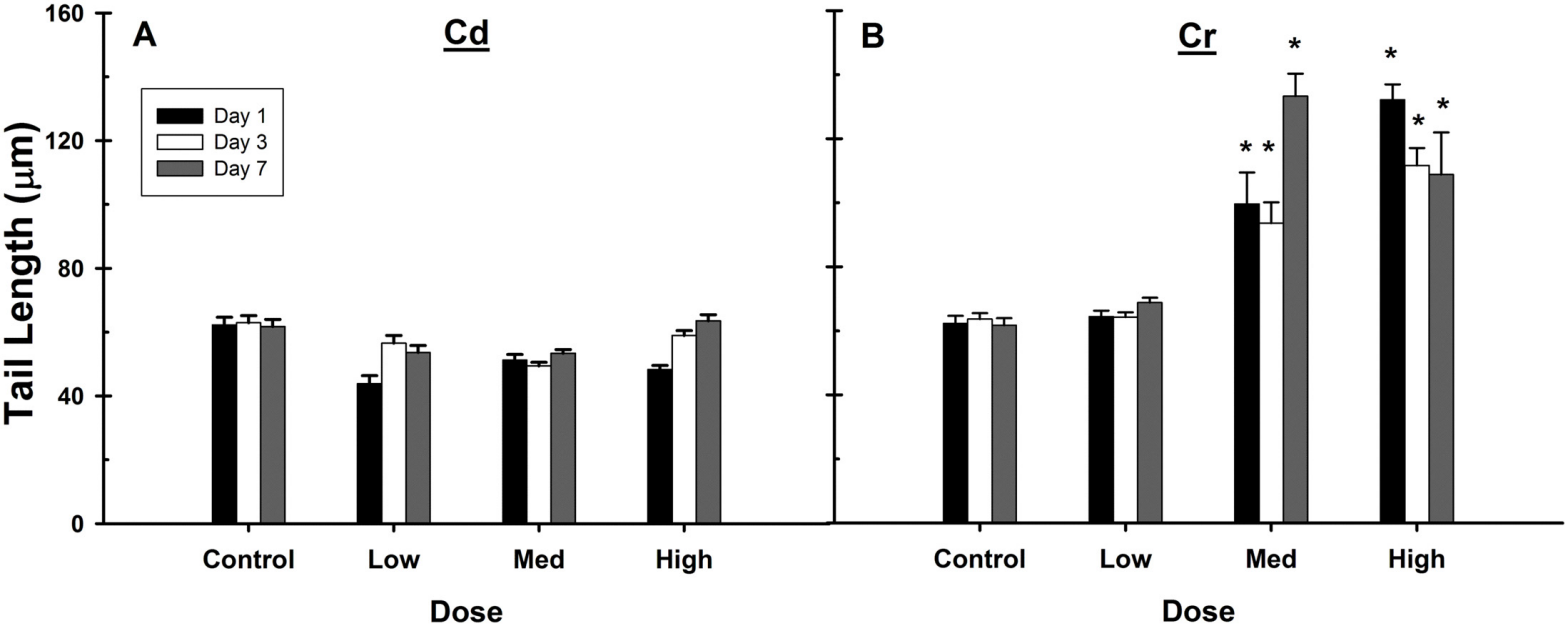
Cr, but not Cd exposure induced DNA damage in rat liver. A comet assay was performed to assess DNA strand breaks in hepatocytes from Cd (A) or Cr (B) treated animals. Cd exposure had no effect on Comet tail length. However, Cr induced DNA strand breaks at the medium and high doses, and this was sustained at all days examined. Data are expressed as mean ±SE, n = 2–7 animals per group. ^†^Only two comet assays were available for analysis on days 1 and 3. *Significantly different from control on each day, p<0.05.

Both Cd and Cr have also been shown in cell culture to be able to induce DNA strand breaks [54,62]. Evidence of DNA strand breaks was observed via a Comet assay after treatment with the medium and high doses of Cr (Fig 5B), which is known to form DNA adducts. No effect was seen in Cd treated livers (Fig 5A), consistent with a previous report that also used an intraperitoneal route of exposure at a higher dose (4 mg/kg) [63].

### Microarray analysis

**Fig 6:**
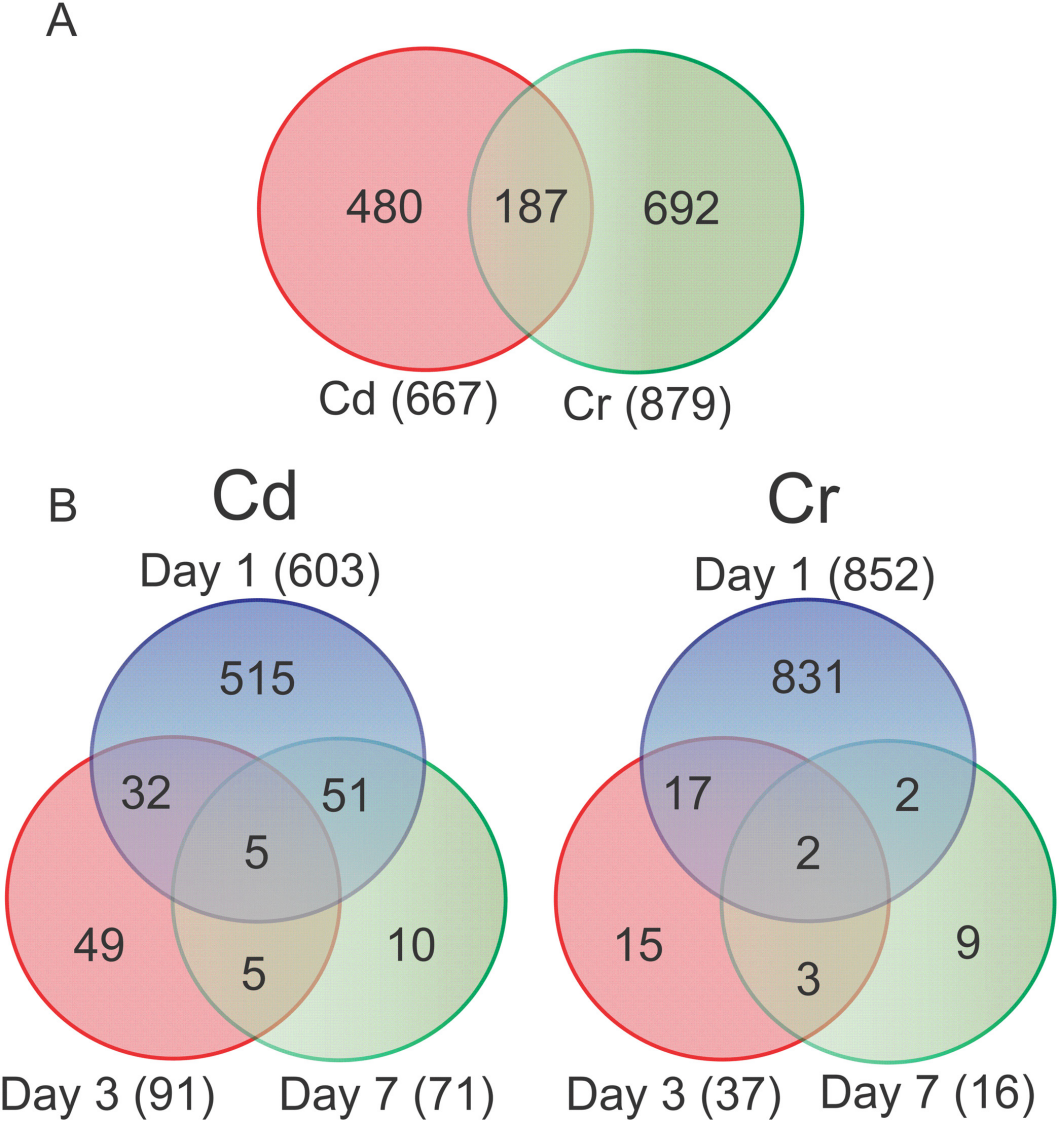
Differentially expressed probe sets and persistent changes in expression following Cd or Cr exposure in the liver. Venn diagrams of (A) total probe sets from all time points for each metal and (B) probe sets on each day included for enrichment analysis. Probe sets were considered significant with a fold-change ≥ 1.8 or ≤ -1.8 and either an FDR < 0.05 (day 1) or unadjusted *p*-value < 0.05 (days 3 and 7).

In order to better understand the cellular response and to gain insight into the mechanism of metal induced damage, microarray analysis was used to identify changes in gene expression. The data was processed using the RMA method and filtered for present genes, and we selected subsets of the genes on each day with a fold-change ≥ 1.8 and a FDR ≤ 0.05. Although we were able to identify a number of genes that were differentially expressed on day 1, there were little or no DEGs identified on days 3 and 7 using this strict criteria. As one of the goals of the study was to examine how genes changed in the liver over time, we used a less stringent set of criteria (fold-change ≥1.8 and unadjusted *p*-value < 0.05) on the samples from days 3 and 7 in order to identify genes and processes that were still affected at these later time points. A total of 667 and 879 unique probe sets were identified over all time points for Cd and Cr, respectively (Fig 6; Table 1). Despite the less stringent criteria, the vast majority of the changes occurred on day 1 for both metals. There were 187 differentially expressed genes after both Cd and Cr exposure, possibly representing a common gene response between the metals, with 178 of these occurring on day 1. Of those identified on day 1, six did not change in the same direction for both metals (*Aldh1a1*, *Ccnd1*, *Rad51*, *Spink1*, *RGD1561849*, and *Mybl1*).

**Table 1:** Differentially expressed probe sets in Cd and Cr exposed livers with each selection criteria.

# Significant Probe sets	Cd			Cr		
	Day 1	Day 3	Day 7	Day 1	Day 3	Day 7
Total	603	56(91)	0(71)	852	0(37)	0(16)
Up-regulated	304	49(79)	0(24)	489	0(25)	0(10)
Down-regulated	299	7(12)	0(47)	363	0(12)	0(6)

Number of probe sets included for further analysis. Probe sets were considered significant with a fold-change ≥ 1.8 or ≤ -1.8 and an FDR < 0.05. Values in parentheses are probe sets included for analysis that met the fold-change cutoff and had an unadjusted *p*-value < 0.05, but did not meet the FDR cutoff.

We performed a functional enrichment analysis in Ingenuity Pathway Analysis (IPA) software using the differentially expressed genes at each time point to identify cellular processes affected by exposure. A pathway was considered significantly enriched with a Fisher’s Exact test *p*-value ≤ 0.05 and including at least 3 genes. The same genes were often found in multiple enriched pathways, which is a bias inherent in any enrichment analysis [64]. There were no enriched pathways after Cr exposure on day 7. The majority of the IPA canonical pathways identified represent six broad categories (Table B in S2 File), including oxidative stress, DNA damage, cell cycle, inflammation, lipid metabolism, and amino-acid metabolism. There were thirteen pathways that were common to both exposures. The majority of the identified inflammation pathways were found after Cd exposure, and pathways were identified at all three time points. The enrichment of IPA canonical pathways unique to each metal exposure was also examined (Table C in S2 File). The first analysis examined the remaining pathways that were not included in the above categories, and yielded three pathways unique to Cd exposure and eight unique to Cr exposure. Next, pathway enrichment was examined using the DEG sets unique to each metal exposure. After filtering out pathways already identified in Table B and the previous analysis, there were an additional three unique pathways for Cd and six pathways for Cr. Unfortunately, these analyses did not reveal any additional insight into the mechanisms of toxicity. The majority of enriched pathways identified could be classified into one of six response categories discussed in this manuscript, while others are enriched for non-specific intracellular signal transduction genes (e.g., G-protein coupled receptor subunits, kinases). The rest of the identified pathways from the enrichment analyses using the exposure specific genes were the same pathways identified in our original analysis. This is an expected finding with enrichment analyses in general and is related to the distribution of differentially expressed genes above and below the significance threshold (i.e., false negatives in one of the chemicals but not the other). The only potentially interesting finding is the enrichment of the Estrogen Biosysthesis and Androgen Biosysthesis canonical pathways after Cr exposure. The rate limiting enzyme in androgen synthesis (Cyp17a1) is up-regulated while genes further down the pathway are down-regulated. This would appear to result in an accumulation of androstenedione. This precursor could feed into the Estrogen Biosynthesis pathway; however, all of the genes identified in this pathway were down-regulated. The biological significance of these observations is unknown.

Functional annotation clustering data using biological process GO terms in DAVID supported the pathway analysis (S2 File). Of the six statistically significant clusters for Cd, two contained inflammation related processes, and one was enriched for cell cycle processes. Of the three significant clusters for Cr, two were enriched for RNA processing and DNA replication. In addition, the DNA replication KEGG pathway was also enriched after Cr exposure.

The activation or inhibition of transcriptional regulators (TRs) was predicted by IPA and used to improve our understanding of the regulatory pathways involved in responses to Cd and Cr toxicity. IPA defines a TR as any molecule that can affect the expression of another molecule, including transcription factors, cytokines, microRNAs, receptors, kinases, chemicals and drugs. Functional activation of the TR was determined by calculating a z-score based on the differential expression of downstream gene targets and comparing their change in expression after exposure to what is known about how activating or inhibiting a particular TR would influence that gene. There were 27 TRs identified after Cd exposure and 17 following Cr exposure, with eight in common for both metals (RELA, MYC, MYCN, BRCA1, IRF1, IRF7, TRIM24, and STAT3; Table 2). In most cases the predicted activation is similar between Cd and Cr, however, MYC related regulators are predicted to be inhibited after Cd exposure and activated after Cr exposure. Besides what was observed on day 1, there is little temporal information in the Cr data, as only STAT5A was predicted to be activated on day 3 and there were none on day 7. Almost half of the significant TRs related to DNA damage or the cell cycle (TP53, MYC, FOXM1, and E2F2) on day 1 are also significant on day 7; however, there were no TRs identified in this category on day 3. This is consistent with the number of common genes differentially expressed between days (Fig 6), where more are in common between day 1 and day 7 than day 1 and day 3. TRs related to inflammation were identified for all days after Cd exposure and on days 1 and 3 after Cr exposure. The activation states of the TRs identified at more than one time point did not change.

**Table 2:** Activation or inhibition of selected transcriptional regulator activity predicted by IPA.

Oxidative stress							
Cd				Cr			
Upstream regulator	Predicted Activation State	Day	z-score	Upstream regulator	Predicted Activation State	Day	z-score
NUPR1	Activated	7	3.16	NFE2L2	Activated	1	2.76
RELA*	Activated	1	2.73	ATF4	Activated	1	2.73
NFKBIA	Activated	1, 3	2.55, 2.54	NFKB1	Activated	1	2.42
FOXO3	Activated	1	2.02	RELA*	Activated	1	2.02
**DNA damage/Cell cycle**							
**Cd**				**Cr**			
Upstream regulator	Predicted Activation State	Day	z-score	Upstream regulator	Predicted Activation State	Day	z-score
TP53	Activated	1, 7	3.56, 2.14	MYC**	Activated	1	3.83
MYC**	Inhibited	1, 7	-3.25, -2.85	HDAC1	Inhibited	1	-2.95
FOXM1	Inhibited	1, 7	-3.25, -2.31	MYCN**	Activated	1	2.49
MYCN**	Inhibited	1	-2.43	HDAC2	Inhibited	1	-2.16
TP73	Activated	1	2.36	BRCA1*	Activated	1	2.07
PML	Activated	1	2.35				
E2F2	Inhibited	1, 7	-2.24, -2.24				
CDKN2A	Activated	1	2.14				
BRCA1*	Activated	1	2.06				
YBX1	Inhibited	3	-2.09				
**Inflammation**							
**Cd**				**Cr**			
Upstream regulator	Predicted Activation State	Day	z-score	Upstream regulator	Predicted Activation State	Day	z-score
IRF7*	Activated	1, 3	5.24, 2.45	STAT3*	Activated	1	2.61
TRIM24*	Inhibited	1, 3	-5.24, -2.45	TRIM24*	Inhibited	1	-2.45
IRF3	Activated	1	4.20	NFIX	Inhibited	1	-2.41
IRF5	Activated	1	3.91	IRF7*	Activated	1	2.39
STAT1	Activated	1	3.65	NCOA2	Activated	1	2.20
IRF1*	Activated	1	3.58	IRF1*	Activated	1	2.15
STAT4	Activated	1, 3	3.33, 2.43	JUN	Activated	1	2.11
FOXO1	Inhibited	7	-2.97	STAT5A	Activated	3	2.00
STAT5B	Inhibited	1	-2.80				
STAT3*	Activated	1	2.79				
STAT2	Activated	1	2.56				
TCF3	Activated	1, 7	2.50, 2.65				
NR1H3	Inhibited	1	-2.45				
NR1H2	Inhibited	1	-2.10				
IGF2BP1	Activated	3	2.00				

Predicted activation or inhibition of activity for selected Transcriptional Regulators (TR) predicted by IPA based on the gene response. A number of transcriptional regulators identified are associated with the pathways in Table B in S2 File. Some regulators may be associated with more than one pathway or stress response, but appear in this table only once.

*The same TR is identified for both metals.

**The same TR is differentially affected between Cd and Cr.

## Discussion

The toxic industrial metals Cr and Cd pose an occupational and environmental exposure risk to both the general population and military personnel. Adverse health effects following acute occupational or accidental exposures of either metal include a range of mild gastrointestinal issues like nausea, vomiting, and diarrhea to more serious effects such as renal or hepatic damage, organ failure, and even death [18,65]. Rats were injected intraperitoneally with CdCl_2_ or Na_2_Cr_2_O_7_, and we examined the responses in the liver at 1, 3, and 7 days post-exposure. ROS levels increased with time post exposure and correlated with markers of lipid peroxidation and DNA damage. Analysis of the transcriptomic responses after Cd and Cr exposure in the liver suggest a persistent inflammatory response may be partially responsible. These insights into the molecular mechanisms of metal toxicity have also revealed potential biomarkers of metal exposure and effect.

### Oxidative stress

The cellular oxidative stress response is affected by a diverse group of environmental and internal stimuli which are characterized by their ability to induce ROS. While Cd indirectly induces ROS by altering the intracellular redox balance via substitution on metalloproteins and glutathione depletion [22,66], Cr induced ROS is primarily due to participation in Fenton-like redox cycling [21]. Both Cd and Cr induced ROS, including •OH and H_2_O_2_ (Figs 2 and 3), which increased with time from exposure. The lack of H_2_O_2_ induction at the early time points (Fig 3) could be due to it being consumed via Fenton-like reactions as Cr(VI) is reduced to Cr(III). Following Cd exposure, the late appearance of ROS may have been the result of an oxidative burst from a sustained inflammatory response and activation of Kupffer cells [56], as evidenced by the persistent up-regulation of inflammation related genes (Fig B in S1 File; Table B in S2 File).

ROS can induce direct cellular injury via radical reactions with proteins, DNA, and lipids. We observed increased lipid peroxidation, after both Cd and Cr exposures, which were sustained for the length of the experiment for most doses (Fig 4). As lipid peroxidation often accompanies rather than causing cell death [59], the data suggests that liver injury is likely occurring at the high dose of Cd and all doses of Cr. Additionally, we can speculate that, at the low dose of Cr, the liver was able recover from the initial insult and regain its membrane integrity.

While the mechanisms of metal-induced generation of ROS may differ, their resulting effects on cell signaling appear to result from a common mechanism [67–72]. Differentially expressed gene lists for both metals were enriched for the same oxidative stress associated pathways, including the prototypical nuclear factor erythroid 2-related factor 2 (Nrf2) pathway. The Nrf2 pathway is highly conserved across vertebrates and controls the expression of an array of antioxidant response element—dependent genes (drug metabolizing enzymes/transporters, antioxidant enzymes/proteins, and oxidant signaling proteins) to regulate the physiological and pathophysiological outcomes of ROS exposure[73]. Classical Nrf2 regulated detoxification genes were affected. For example, the NAD(P)H:quinone oxidoreductase-1 (*Nqo1*) gene was up-regulated by Cd and Cr, while heme oxygenase-1 (*Hmox1*) was induced by Cd.

Exposure to both metals increased the expression of MT genes *Mt1a and Mt2a*. MT gene levels remained elevated out to day 7 after Cd exposure, while after Cr exposure there was a biphasic response, as levels were elevated on days 1 and 7 but were at control levels on day 3. The initial increase in MT expression is likely a response to the Cr itself. Cr levels are highest on day 1 and have dramatically decreased by day 3 (Fig 1) and the expression of MT genes follows this same trend. The secondary induction on day 7 may be occurring in response to the presence of redox active metals, such as Fe and Zn, released from damaged metalloproteins, which could also account for the increases in H_2_O_2_ and •OH observed.

GSH plays an important role in ROS detoxification, and GSH-related genes are also known to be regulated by Nrf2 [74]. Surprisingly, *Gclc* (a subunit of the rate-limiting enzyme in GSH synthesis) as well as multiple glutathione-S transferase genes were either unaffected or down-regulated in our experiment. This apparent contradiction may have been due to our sample collection times, as *Gclc* gene and protein levels peak in other cell types around 6–12 h after Cd exposure *in vitro* [75], whereas our first sample was not taken for 24 h. Additionally, when p53 is activated by oxidative stress and DNA damage, as is seen in our data, it has been reported to suppress Nrf2-mediated transcription [76]. The reduction in cellular GSH related genes may also reflect the induction of apoptosis in the hepatocytes, as the release of cellular GSH and the creation of a more oxidized intracellular environment have been demonstrated to be an important step in the induction and/or progression of apoptosis [77–79].

### Lipid and amino acid metabolism

The enrichment of pathways related to lipid and amino-acid metabolism are likely to represent the cell’s response to energy deprivation and protein damage due to oxidative stress. Many of the same pathways in the Amino Acid Metabolism category enriched in DEG lists for Cd exposure were also identified after Cr exposure, suggesting a common stress response. For example, all of the genes identified in the Superpathway of Citrulline Metabolism (*Ass1*, *Oat*, *Gls2* after both Cd and Cr exposure and *Prodh* and *Otc* after only Cr exposure) were down regulated early, but returned to control levels at later time points. These genes participate in amino acid degradation and the urea cycle, leading to an increase in amino acids available for protein synthesis [80]. Additionally, the decrease in *Otc* expression would allow for a shunting of carbamoyl phosphate from the urea cycle to the synthesis of pyrimidines for *de novo* nucleotide synthesis for DNA repair [81]. Genes from the TR/RXR Activation pathway involved in gluconeogenesis and lipogenesis (*G6pc*, *Me1*, and *Spot14*, as well as *Pc* for Cr), were also down-regulated, which would be expected to result in an increase in ATP synthesis via glycolysis or the generation of reducing power via NADPH in the pentose phosphate pathway [82]. Common to a majority of the lipid metabolism pathways identified is the increased expression of cytosolic aldehyde dehydrogenase genes (*Aldh1a1* and *Aldh1a7*) after Cr exposure. These genes play a major role in the detoxification and cellular defense against oxidative damage induced by reactive lipid aldehydes [83,84]. A similar increase in aldehyde dehydrogenase genes was not observed after Cd exposure as there was not lipid peroxidation present at the dose selected for microarray analysis.

### DNA damage

The DNA damage response is a network of interacting signal transduction pathways, consisting of sensors, transducers and effectors, which together detect DNA lesions, signal their presence and promote their repair [85]. DNA damage can be caused by ROS arising from multiple sources, such as oxidative respiration, redox-cycling mediated by metals, or inflammatory processes mediated by macrophages and neutrophils. ROS can interact with DNA, leading to adduct formation that impairs base-pairing and/or blocks DNA replication and transcription, potentially leading to base loss and single or double DNA-strand breaks [86].

Both Cd and Cr have been reported to induce DNA damage and cell cycle arrest [16,54,55,62,87–89], and our data support these findings. DNA damage-related pathways were enriched at all time-points after Cd exposure, although we were unable to detect DNA strand breaks. A majority of the DEGs in these pathways appear to be down regulated following Cd exposure at both early and late time points (Fig B in S1 File), while more are up-regulated after Cr exposure. The enrichment of DNA damage and cell cycle regulation-associated pathways we observed are consistent with the *in vitro* and *in vivo* Cd-induced DNA damage reported by other groups [16,62,87–91].

Chromium has been shown to directly interact with DNA and cause damage by forming DNA adducts and causing DNA strand breaks via ROS induction [24,92]. A number of our observations after Cr exposure were consistent with previous *in vitro* exposures done in our lab [25]. Multiple genes involved in the pre-replication complex (*Mcm4*, *Mcm5*, *Mcm6*, *Mcm7*, *Cdt1*), which plays an important role in DNA replication and repair, are up-regulated after Cr exposure. These genes encode subunits of the minichromosome maintenance complex, which acts as a helicase in DNA replication. In addition, other genes involved in DNA replication and repair, such as replication protein A (*Rpa2*), flap structure-specific endonuclease 1 (*Fen1*), DNA polymerase ε (*Pole3*), and DNA polymerase δ (*Pold2*) are also up-regulated as a result of exposure to Cr, suggesting that there is an increase in DNA synthesis to repair the Cr-induced DNA damage or to allow for increased proliferation to replace damaged hepatocytes. We did not observe the induction of genes related to base or nucleotide excision repair. However, DNA strand breaks can also induce homologous combination repair mediated through RAD51 and BRCA1 [93]. In our data, *Rad51* message is up-regulated and the Brca1 TR is predicted to be activated, indicating that repair of Cr-mediated DNA damage is likely proceeding through the homologous recombination repair pathway.

### Cell cycle

The cell cycle is the highly ordered mechanism by which cells replicate their DNA and divide. The expression of cyclins and their associated cyclin dependent kinases control progression through the cell cycle phases of G1, S, G2, and M to allow for cell replication [94]. Hepatic injury and the release of pro-inflammatory signals can stimulate hepatocytes to leave a quiescent state (G0) and undergo rapid proliferation to replace dead cells [95]. Cell cycle checkpoints are present at the transitions from G1 to S and from G2 to M to help ensure the accuracy of DNA replication and division. ROS-induced cellular and DNA damage can lead to cell cycle arrest at these checkpoints to allow for repair or removal of the cell via apoptosis.

Both metals have been reported in multiple model systems to halt cell division at both the G1/S and G2/M checkpoints [87–89,96,97], indicating there are likely cell type and or dose-dependent effects influencing where the cell cycle is halted following a toxic exposure. Our analysis identified pathways and TRs associated with cell cycle regulation (Table 2 and B). Interestingly, the G2/M checkpoint was identified after Cd exposure and the G1/S checkpoint was identified after Cr exposure while the GADD45 pathway, which is known to play an important role in the G2-M checkpoint [98–100], was identified following both exposures. The G2/M enrichment induced by Cd is likely a p53 dependent process. The TR p53, which was predicted to be activated in our data, appears to suppress the G2/M transition by negatively regulating the expression of cyclin B1, CDK1, and topoisomerase II alpha [101,102]. In fact, all three of these genes (*Ccnb1*, *Cdk1*, and *Top2a)* were down-regulated in our data, suggestive of a G2/M arrest. After Cr exposure we observed an early induction of *Cdkn1a* (p21), whose activation leads to a stop in the progression through the G1/S checkpoint [103]. *Cdkn1a* is regulated by p53, a transcription factor that can be activated by a number of intrinsic factors such as DNA damage and oxidative stress, and is a major regulator of cell fate. p53 can activate DNA repair mechanisms, inducing cell cycle arrest, or it can initiate apoptosis to remove cells which cannot be repaired. It is likely that Cr-induced •OH is activating p53 [104] leading to p21 induction and G1 arrest.

### Inflammation

There is a critical balance between cellular oxidants and antioxidant defenses which cells must maintain. Disruption of this balance can result in a chronic inflammatory state, leading to damage to the cells involved and to the surrounding tissue due to activation of signaling pathways, inflammatory cytokine production, altered gene expression, and other cellular modifications [51]. Inflammation is usually a protective response of an organism to injury or infection, whose role is to eliminate injury-inducing agents, prevent tissue damage and/or initiate repair processes and restore physiological functions of the affected tissue [105]. It is mediated via multiple secreted and membrane bound factors produced by cells participating in the inflammatory process either directly (e.g., an injured, necrotic, or apoptotic cell or surrounding tissue) and/or responding to the inflammatory stimulus, which can amplify the tissues’ response and affect the course of the inflammatory episode [105]. In our data, we identified multiple inflammation pathways and TRs (Table 2 and B) that were affected well after the exposure occurred.

Cd toxicity has been linked to inflammation, as there is often an infiltration of inflammatory cells after acute exposures [106–108]. Multiple genes from inflammation related pathways were differentially expressed in our data sets. The up-regulation of these genes appeared to persist after Cd treatment, and mostly return to control levels after Cr exposure (Fig B in S1 File). As would be expected after an insult to the liver, the pathways enriched generally appear to flow from an initial innate immune response to a more adaptive or recovery-like response.

ROS-induced lipid peroxidation generates electrophilic molecules that can bind to and modify proteins that trigger pro-inflammatory responses through binding to cellular pattern recognition receptors [109]. The sustained lipid peroxidation seen here (Fig 4) may therefore be contributing to the generation of a sustained immune response, as illustrated by the enrichment in inflammation related pathways. Receptor activation can lead to the activation of Kupffer cells, the resident macrophages in the liver, in response to endothelial and parenchymal cell injury, resulting in a secondary inflammatory response. The activated Kupffer cells release additional inflammatory mediators to recruit neutrophils, and together can then release other cytotoxic mediators, such as ROS, reactive nitrogen species, bioactive lipids, and hydrolytic enzymes to cause further liver injury [106]. This type of secondary inflammation may be the source of the increased ROS we observed on days 3 and 7 post Cd exposure.

Multiple inflammation-related TRs, such as STAT3, IRF1, IRF7, and TRIM24 were activated or inhibited, and their activation state is the same after exposure to either metal. The transcription factors STAT3, IRF1, and IRF7 are mediators of the acute phase inflammatory response [110,111]. STAT3 may also protect against liver injury [112]. TRIM24 is a negative regulator of IRF and STAT signaling [113], and its inhibition at multiple time points is consistent with a sustained inflammatory response.

Genes for a number of cytokines and chemokines were up-regulated in response to Cd and Cr exposure (*Cxcl1*, *Cxcl9*, *Cxcl10*, *Ccl6*, *Ccl9*, *and Ccl27*), which could result in the recruitment of inflammatory cells to the liver. *Cxcl1* was up-regulated early after exposure to both metals, but remained up-regulated at later time points only after Cd exposure. Other genes associated with inflammation are also up-regulated (*Lcn2*, *S100A8*, *S100A9*, and *Fas*). *Lcn2*, which is elevated at both early and late time points, is an acute-phase protein that appears rapidly in the bloodstream in response to a systemic infection or tissue injury and appears to be hepato-protective [114]. S100A8 and S100A9 proteins can act as chemokines or damage signals and are constitutively expressed in neutrophils, monocytes, and dendritic cells, and can also be induced in other cell types such as mature macrophages [115]. They are both significantly induced early and may reflect inflammatory cell infiltration of the liver. This may be more relevant after Cd exposure as their increased expression persists for the duration of the experiment, whereas their expression has returned to near control levels on days 3 and 7 after Cr treatment. The death-receptor Fas (CD95) can also induce pro-inflammatory cytokine release from macrophages [116]. Taken together, the increased expression of these genes we observed support our hypothesis that both Cd and Cr exposure can induce a sustained inflammatory response.

### Biomarkers

**Fig 7:**
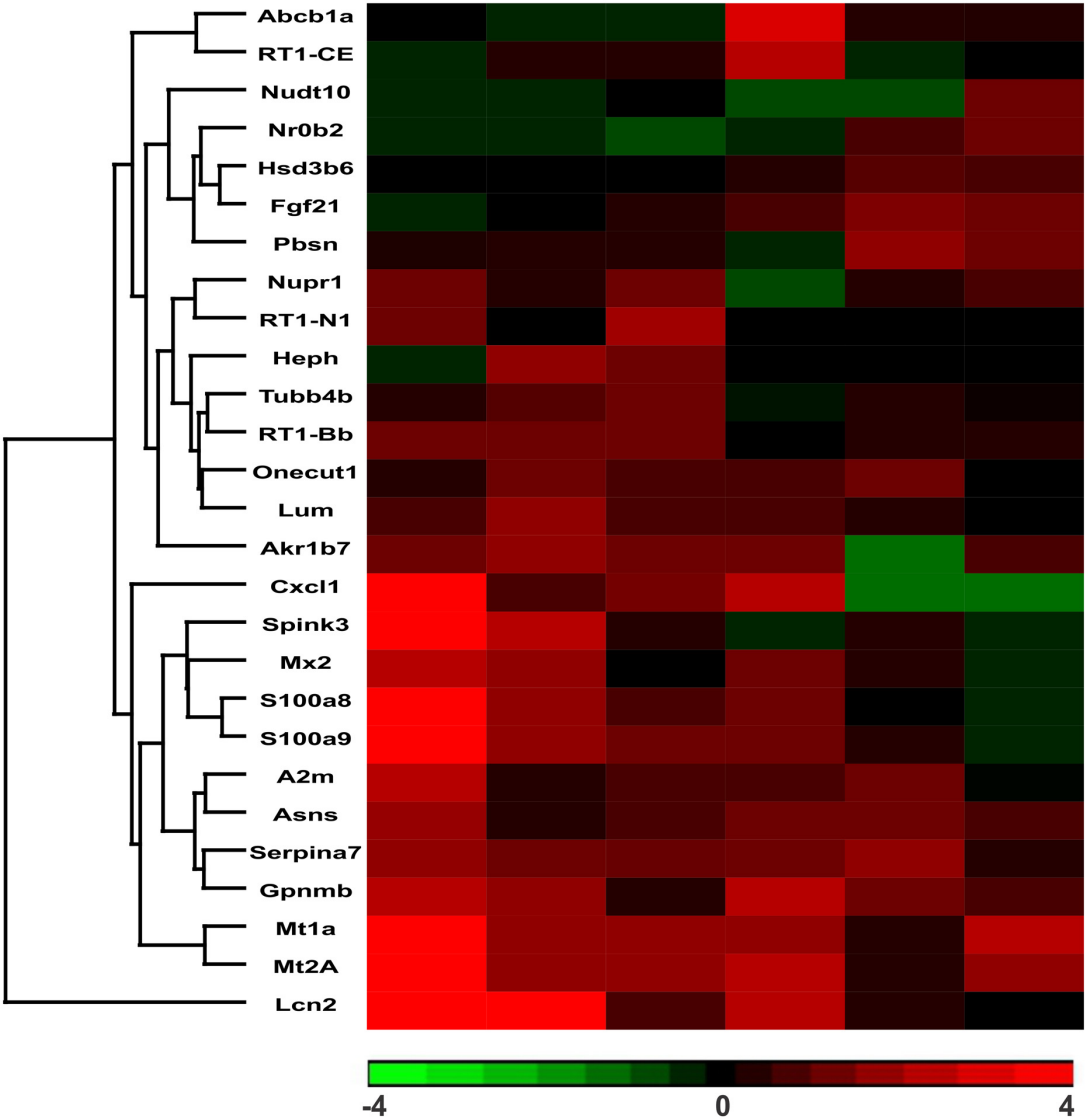
Time-dependent changes in candidate biomarker gene expression. The figure shows hierarchical clustering of the top 5 up-regulated DEGs on each day for each metal. When the same gene was one of the top genes for a different day of the same metal the next highest up-regulated gene was included for that day. The values shown in the heat map are the log_**2**_ ratio of change of the genes compared to the unexposed controls.

One of the goals of these experiments was to identify candidate biomarkers of metal exposure and toxicity in the liver. We examined the temporal expression of the top 5 up-regulated genes for each metal as potential candidates (Fig 7). Potential candidates of metal exposure include *Cxcl1*, *S100a8* and *9*, *Mt1a*, *Mt2a*, and *Lcn2*, which showed a high degree of up-regulation on day 1. Many of these genes remained elevated at later time points as well. Some genes displayed unique expression patterns for each metal. For example, *Abc1a* and *RT1*-CE, a multidrug efflux transporter and major histocompatibility protein respectively, were up-regulated early after Cr exposure, but were unaffected by Cd at the same time point. In contrast, *RT1-N1* and *Heph* were up-regulated after Cd exposure but unaffected by Cr. *Heph*, *Tubb4*, *Fgf21*, *NrOb2* are representative of potential late indicators of exposure as they were minimally or unaffected on day 1, but increased in expression on days 3 and 7. We also observed an up-regulation of genes that have been identified as markers of tissue injury [117–119]. These include *Lcn2*, *Lgals3*, *Gpnmb*, *Hgf*, and *Timp1* after exposure to either Cd or Cr, as well as an increase in *A2m* expression after Cd exposure. Some are also already utilized in liver fibrosis screening panels [117]. These genes appear to represent a common stress response that is induced in multiple tissues after injury. This type of result can hinder efforts to develop specific biomarkers of toxicant exposure, but could narrow the field of available genes to be used for the development of biomarkers to predict tissue injury after toxic exposures.

## Conclusion

There is little information in the literature about the effects of metal-induced ROS, its’ associated cellular damage, and the inflammatory response in the liver after a single acute exposure. The few studies that we have identified do not examine beyond a few days [50,120,121]. This study has provided insight into the mechanism of Cd and Cr induced liver damage and has provided experimental evidence for the contribution of •OH in their toxicity. In addition, this data provides insight into the temporal effects on gene expression after metal exposure. We demonstrated that both Cd and Cr exposure resulted in a delayed induction of ROS in the liver, a majority of which can be attributed to the formation of •OH. The appearance of cellular damage markers mirrors the induction of ROS, with the majority of the induction occurring at higher doses and later time points. In contrast to the ROS induction, microarray analysis revealed a large early effect on gene expression after both Cd and Cr exposure that tended to decrease over time. Differing patterns in gene expression were observed after Cd and Cr exposure, although there was a subset of genes whose expression changes persisted well after exposure. Pathway enrichment analysis identified major stress response pathways as indicators of metal toxicity. The delayed ROS induction after Cd exposure is likely the result of a secondary inflammatory response, as evidenced by the enrichment of persistent inflammation related genes and pathways, while the increased ROS after Cr exposure is more likely to result from the release of other Fenton-cycling metals from damaged metalloproteins or Cr itself. Both Cd and Cr induced metal specific and shared gene responses which were used to propose candidate biomarkers. Both early and late indicators of exposure and effect were identified for each metal, as well as more general markers of exposure. Future work should utilize histopathological techniques to confirm inflammatory cell infiltration after Cd exposure as well as validate identified candidate biomarkers.

## Supporting Information

S1 File Figures A and B showing results of qPCR used for gene array dose selection and heat maps of DEG expression over time, and Table A comparing fold-change values from qPCR to microarray results. (DOCX)

S2 File DEG lists, including fold-change values, for each metal and day, Tables B and C, the present call lists, and results from DAVID used in this study. (XLSX)
