# Supplementary material for: Temporal Changes in Rat Liver Gene Expression after Acute Cadmium and Chromium Exposure
Source: PLoS One. 2015 May 19;10(5):e0127327. doi: 10.1371/journal.pone.0127327 (PMC4437902; doi:10.1371/journal.pone.0127327)
Supplement: S1 File — (DOCX) [file pone.0127327.s001.docx]

**
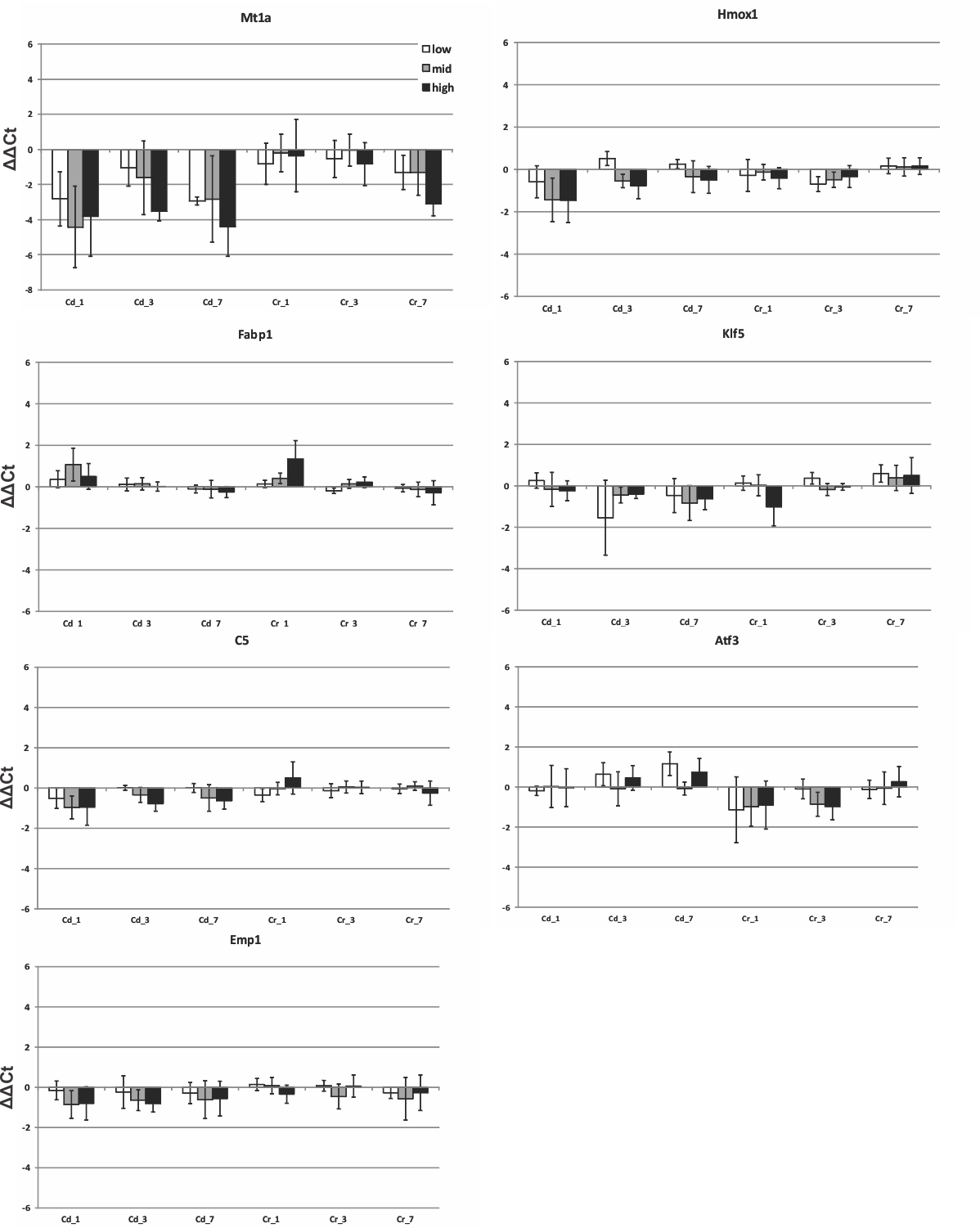
Figure A. ΔΔCt data used for microarray dose selection**. Three liver samples were randomly selected for each condition and total RNA extracted using Trizol solution per the manufactures instructions. SYBR Green based qPCR was performed as previously described (Permenter et al., 2011).

Permenter MG, Lewis JA, Jackson DA. Exposure to nickel, chromium, or cadmium causes distinct changes in the gene expression patterns of a rat liver derived cell line. *PLoS One*. 2011;6(11):e27730.


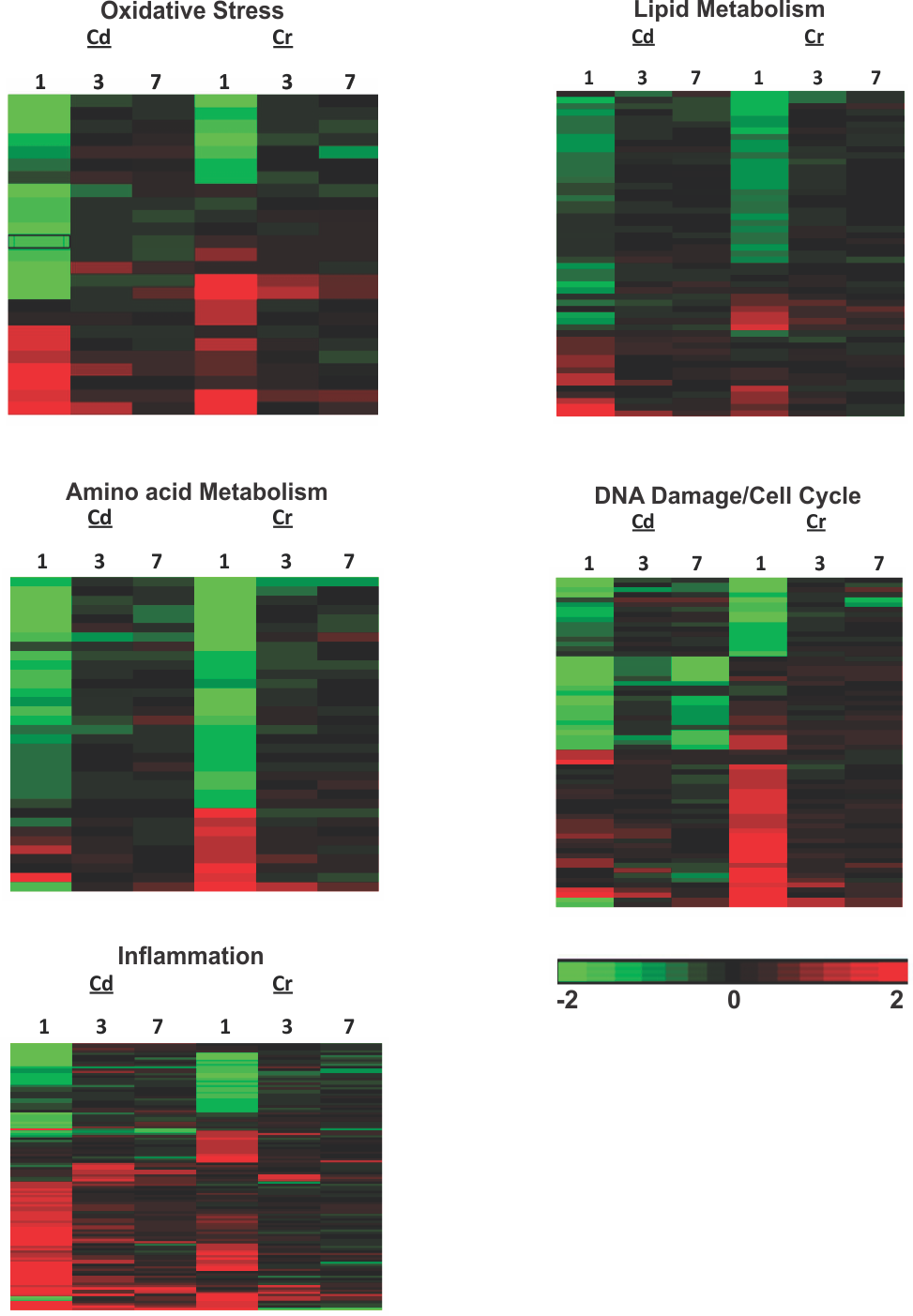


**Figure B. Time-dependent changes gene expression.** The figure shows hierarchical clustering of the genes that were enriched in the IPA canonical pathways (Table 2) on each day for each metal. The values shown in the heat map are the log_2_ ratio of change of the genes.

|  |  | **Cd Mid** | | | **Cr High** | | |
| --- | --- | --- | --- | --- | --- | --- | --- |
|  | Day | 1 | 3 | 7 | 1 | 3 | 7 |
| ***Atf3*** | Array | 1.30 | 1.05 | -1.04 | 2.57 | 1.74 | -1.04 |
|  | qPCR | -1.02 | 1.06 | 1.05 | 1.87 | 1.97 | -1.20 |
| ***C5*** | Array | 1.71 | 1.27 | 1.14 | -1.10 | 1.01 | 1.03 |
|  | qPCR | 1.95 | 1.27 | 1.41 | -1.41 | -1.02 | 1.19 |
| ***Emp1*** | Array | 1.74 | 1.62 | 1.20 | 1.35 | 1.27 | 1.01 |
|  | qPCR | 1.81 | 1.56 | 1.53 | 1.27 | -1.04 | 1.20 |
| ***Fabp1*** | Array | -1.35 | 1.01 | -1.03 | -1.14 | -1.04 | 1.01 |
|  | qPCR | -2.10 | -1.10 | 1.08 | -2.52 | -1.17 | 1.22 |
| ***Hmox1*** | Array | 2.41 | 1.61 | 1.27 | 1.38 | 1.13 | -1.04 |
|  | qPCR | 2.71 | 1.46 | 1.27 | 1.33 | 1.27 | -1.11 |
| ***Mt1a*** | Array | 12.74 | 3.69 | 3.87 | 3.27 | 1.14 | 5.06 |
|  | qPCR | 21.46 | 3.07 | 7.10 | 1.28 | 1.79 | 8.50 |

**Table A. Comparison of fold-change values between microarray and qPCR for select genes.** The fold-change in gene expression was compared between the qPCR data and our microarray data. As expected, there is a good correlation between qPCR gene expression values and microarray platform results (r^2^ = 0.87). Furthermore, for every condition where the microarray identified a change of at least 1.5 fold, the qPCR confirmed a fold change of at least 1.5 in the same direction. Potential sources of variability between the two methods likely arise from differences in probe sequence and target location, as well as the limited sensitivity of microarray platforms for detecting weakly expressed genes (Canales et al., 2006). Data pairs for genes which changed 1.5 fold in the array data have been highlighted in red. SYBR Green based qPCR was performed as previously described (Permenter et al., 2011). Fold-change was calculated using the ΔΔCt method.

Canales RD, Luo Y, Willey JC, Austermiller B, Barbacioru CC, Boysen C, Hunkapiller K, Jensen RV, Knight CR, Lee KY, Ma Y, Maqsodi B, Papallo A, Peters EH, Poulter K, Ruppel PL, Samaha RR, Shi L, Yang W, Zhang L, Goodsaid FM. Evaluation of DNA microarray results with quantitative gene expression platforms. *Nat Biotechnol*. 2006 Sep;24(9):1115-22.

Permenter MG, Lewis JA, Jackson DA. Exposure to nickel, chromium, or cadmium causes distinct changes in the gene expression patterns of a rat liver derived cell line. *PLoS One*. 2011;6(11):e27730.
